# *Byrsonima crassifolia*: Ethnobotany, Phytochemistry, and Pharmacology – An Update

**DOI:** 10.1002/cbdv.71756

**Published:** 2026-09-27

**Authors:** João de Jesus Oliveira Junior, Débora Luana Ribeiro Pessoa, Marilene Oliveira da Rocha Borges

**Affiliations:** ^1^ Graduate Program in Biotechnology Northeast Biotechnology Network (RENORBIO) Federal University of Maranhão São Luís Maranhão Brazil; ^2^ Special Coordination of Physiological Sciences and Pathology Center for Biological and Health Sciences Federal University of Maranhão São Luís Maranhão Brazil

**Keywords:** antidiabetic activity, anti‐inflammatory activity, antioxidant activity, *Byrsonima crassifolia*, phytochemistry, traditional medicine

## Abstract

*Byrsonima crassifolia* is a fruit‐bearing species widely distributed across tropical regions of the Americas with potential health applications. This review updates the knowledge base on its traditional uses, phytochemicals, and pharmacological activities to support future research.​ Native communities employ it as a key food and artisanal resource. As a medicinal plant, it has a history of treating diarrhea, diabetes, inflammation, and other conditions.​ Extracts and fractions, mainly from leaves and fruits, reveal numerous bioactive compounds, particularly non‐flavonoid polyphenols, flavonoids, and terpenoids. In vivo and in vitro evidence shows convergence between traditional medicine and a range of pharmacological activities, including antimicrobial, anti‐inflammatory, hypoglycemic, and gastroprotective effects; other reports indicate antioxidant, neuromodulatory, photoprotective, chemopreventive, and antitumor properties.​ Various studies link these pharmacological findings to the antioxidant profile of polyphenols identified in the species. Additionally, individual terpenoids isolated from seeds show promising effects on infection, inflammation, and glycemic control.​ Collectively, these findings indicate that the use of *B. crassifolia* and its bioactive compounds has a scientific basis. However, future research must advance in extract standardization, chemical marker quantification, molecular mechanism exploration, toxicity testing, and clinical trials to validate its therapeutic potential.​

## Graphical abstract

Current knowledge on *B. crassifolia* is integrated, linking its phytochemical diversity with reported pharmacological activities and highlighting the therapeutic potential of this medicinal species.

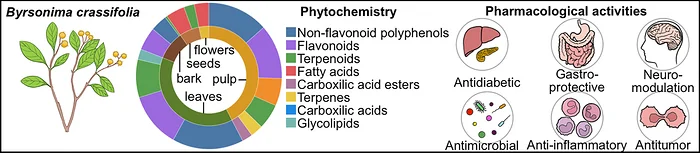

## 1 Introduction

Medicinal plants are valuable sources of bioactive compounds that contribute to the development of new drugs. Pharmacologically relevant plant species represent important genetic resources and contain molecules with unique structures and biological activities often absent in other natural sources [1, 2].

In this context, Brazilian biomes represent promising sources for the bioprospecting of pharmacologically relevant species. The Cerrado, the country's second‐largest biome, harbors a wide diversity of plant species with therapeutic potential, for which anti‐inflammatory, antinociceptive, wound‐healing, antimicrobial, antioxidant, antimutagenic, and antitumor activities have been reported [3, 4, 5]. These properties are attributed to the presence of secondary metabolites, mainly phenolic compounds, flavonoids, and triterpenes, along with coumarins, tannins, steroids, saponins, and reducing sugars (Mendonça et al., 2019) [3].

Among the Cerrado species, *Byrsonima crassifolia* (L.) Kunth stands out due to its traditional use and diverse phytochemical profile [6]. Besides its ornamental use and the economic importance of its fruits for consumption and commercialization [7, 8], this species plays a significant role in traditional medicine, primarily employed to treat gastrointestinal disorders, inflammations, and wounds [9]. The growing scientific interest in *B. crassifolia* has been driven by experimental studies demonstrating diverse biological activities, including antioxidant, anti‐inflammatory, antimicrobial, wound‐healing, and chemopreventive effects [10, 11]. These findings broaden the scope of the species’ potential therapeutic applications and emphasize its value as a source of bioactive compounds for further pharmacological investigation. However, information regarding its ethnobotanical uses, phytochemistry, pharmacological activities, toxicity, and translational potential remains fragmented across the literature.

Previous reviews have addressed the phytochemical and pharmacological aspects of the Malpighiaceae family and the genus Byrsonima [6, 12, 13], or the botanical, nutritional, phytochemical, and health‐related properties of *B. crassifolia* [11]. The present review builds upon these studies by integrating the available evidence on the botanical characteristics, ethnobotany, phytochemistry, pharmacology, toxicology, and future research perspectives of *B. crassifolia*. In addition, it provides an organized catalog of the phytochemicals identified in *B. crassifolia*, classified according to their chemical classes, subclasses, and occurrence in different plant organs, thereby offering an integrated reference for future phytochemical and pharmacological research.

Therefore, this review aims to update and integrate current knowledge on the ethnobotany, phytochemistry, pharmacology, and safety of *B. crassifolia* to support further investigation of its phytotherapeutic potential.

## 2 Methods

This narrative review was based on a literature search conducted up to March 2026 in PubMed, Scopus, Web of Science, SciELO, and Google Scholar. The databases were searched for original articles published in English or Portuguese using the terms “*B. crassifolia*” OR “murici”, followed by complementary searches using keywords covering ethnobotanical, phytochemical, and pharmacological aspects, such as “ethnobotany”, “traditional use”, “phytochemistry”, “phytochemical”, “antioxidant”, “anti‐inflammatory”, “antimicrobial”, “wound healing”, “gastric”, “oxidative stress”, “diabetes”, and “cancer”. Original studies addressing the botanical, ethnomedicinal, phytochemical, nutritional, or pharmacological aspects of *B. crassifolia*, as well as studies published under taxonomic synonyms currently recognized by authoritative botanical databases, were included. Reference lists of retrieved original and review articles were also screened to identify additional relevant studies. Taxonomic nomenclature and synonymy were verified using the botanical databases Flora e Funga do Brasil, Plants of the World Online, World Flora Online, and Tropicos, which were also consulted to provide contextual background. Studies on other Byrsonima species not recognized as taxonomic synonyms of *B. crassifolia* were excluded from the review.

## 3 Botanical Characteristics

The Malpighiaceae family comprises 75 genera and 1,499 species of flowering plants, including trees, shrubs, subshrubs, and lianas, occurring mainly in tropical and subtropical regions. The bulk of family diversity is concentrated in the Americas, which harbor 55 genera and 1,274 species [14]. Within this family, the genus Byrsonima is the most studied, with about 150 described species, among which *B. crassifolia* is the most commonly found in Brazil [11]. The species was first described by Carl Linnaeus as *Malpighia crassifolia* [15]. Subsequently, Karl Kunth transferred the species to the genus *Byrsonima* as *B. crassifolia* [16].

The species has several taxonomic synonyms and common names [17, 18, 19], as shown in Table 1. Notably, *B. fagifolia* Nied., a name used in several phytochemical and pharmacological studies included in this review, is currently recognized as a heterotypic synonym of *B. crassifolia* (L.) Kunth [18, 20, 21].

**TABLE 1:** Scientific synonymy and common names of *Byrsonima crassifolia* (L.) Kunth.

Taxonomic synonyms	Common names
*B. crassifolia* Steud.	Portuguese (Brazil): murici, muruci, muruci‐do‐campo, muruci‐da‐praia, canjicão. Spanish: nance, nanche, chaparro de chinche, chaparro de sabana, chaparro peralejo, chaparro manteco indano, cimarrón, crabo, manteco, manteco sabanero, manero, marushi, maricas, maricao, nance blanco, nancite, nancito, peralejo blanco, peralija, changugo, tapal, yoco. English: golden spoon. French: maurissi, moureiller des Caraïbes, and moureil.
*B. crassifolia* Lunan ex Griseb.	
*B. crassifolia var. cinerea* (Poir.) Nied.	
*B. crassifolia var. crassifolia*	
*B. crassifolia fo. cubensis* (A. Juss.) Nied.	
*B. crassifolia fo. ferruginea* Griseb.	
*B. crassifolia fo. ferruginea* (Kunth) Nied.	
*B. crassifolia subsp. insulata* Cuatrec.	
*B. crassifolia var. jamaicensis* Urb. & Nied.	
*B. crassifolia fo. kunthiana* Nied.	
*B. crassifolia var. lanceolata* Cuatrec.	
*B. crassifolia var. lanceolata* (Poir.) Griseb.	
*B. crassifolia var. moureila* (Aubl.) DC.	
*B. crassifolia var. peruviana* (A. Juss.) Nied.	
*B. crassifolia var. spruceana* (Nied.) Nied.	
*B. cinerea* (Poir.) DC.	
*B. coriacea* (Sw.) DC.	
*B. fagifolia* Nied.	
*B. rhombifolia* A. Juss.	

**FIGURE 1:**
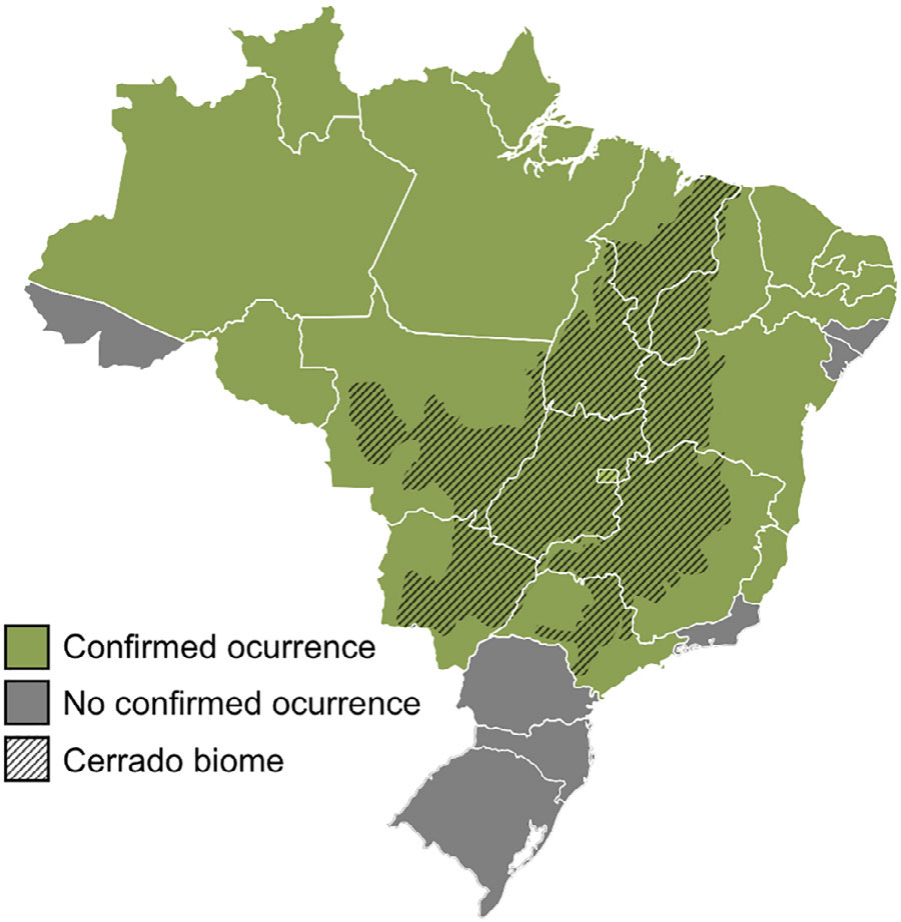
Geographic distribution of *Byrsonima crassifolia* (L.) Kunth in Brazil, based on occurrence data compiled from Flora e Funga do Brasil [14] and published literature [7, 17].

*B. crassifolia* may occur as a shrub or small‐ to medium‐sized tree, 1.5 to 7 m tall. Its trunk is tortuous, with thick bark, and its elliptical leaves measure 6 to 12 cm in length by 2.5 to 7.5 cm in width. The flowers have yellow petals, and the fruits, which are drupes, are globose, 6 to 10 mm in diameter, with a yellow epicarp when ripe [18]. The species' geographic distribution in Brazil covers almost the entire national territory (Figure 1), except for the states of Acre, Alagoas, Sergipe, and Rio de Janeiro, as well as the South Region. *B. crassifolia* occurs in the Amazon, Caatinga, Cerrado, Atlantic Forest, and Pantanal biomes [7, 18]. In addition to Brazil, the species is native to all countries bordering the Brazilian Amazon, as well as Mexico and various countries in Central America and the Caribbean [22]. Accurate botanical identification is a fundamental requirement to ensure the authenticity of plant material and the reproducibility of research involving medicinal plants [23].

## 4 Ethnobotanical and Socioeconomic Importance

### 4.1 Historical and Ethnomedicinal Context

Paleoethnobotanical evidence, such as carbonized seeds, charcoal, and microtraces in human teeth, indicates that *B. crassifolia* may have been used for medicinal purposes in pre‐colonial Mesoamerica between 350 and 900 A.D. [24]. Ethnobotanical studies demonstrate that different parts of the plant are prepared in various forms for medicinal use, with bark being the most frequently cited (Table 2).

**TABLE 2:** Ethnobotanical applications of *Byrsonima crassifolia* (L.) Kunth.

Category	Plant part	Mode of use	Indications	References
Medicinal	B	Topical	Wounds	[25]
	B, L	Oral	Dermatitis	[26]
	B, IB	NR	Wounds	[9]
	B	Oral	Diarrhea	[25]
	B, L	Oral		[26]
	B	Oral		[27]
	B, IB	Oral		[9]
	B	Oral	Stomach pain, diarrhea	[28]
	L, Fl	NR	Pharyngitis, influenza	[29]
	B, L	Oral	Cough	[26]
	B	NR	Pharyngitis	[9]
	B	NR	Kidney inflammation	[9]
	NR	Oral	Menorrhagia	[30]
	B	Sitz bath	Uterine inflammation	[25]
	B	NR		[9]
	B	Vaginal douche	Vaginal infection	[25]
	B	Oral	Snakebite	[31]
	B, L	Oral		[26]
	B, Fr	Oral	Diabetes	[32]
	B, IB	NR	General inflammation	[9]
	B	Chewing	Helminthic infections	[28]
	B	Topical	Umbilical bleeding in newborns	[28]
Food	Fr	Oral	Nutrition	[9]
Fuel	S	Burning	Cooking (firewood, charcoal)	[9]
Handicraft	B	Processing	Dyeing	[9]

B, bark; IB, inner bark; L, leaves; Fl, flowers; Fr, fruits; S, stem; NR, not reported.

### 4.2 Medicinal Uses of Stem Bark

Indigenous communities in Mesoamerica and South America cite *B. crassifolia* among numerous medicinal plants for treating gastrointestinal disorders. In the Mixe indigenous community, the species was classified as likely effective against diarrhea because of its high tannin content, although pharmacological evidence was lacking at the time [27]. Various medicinal applications of *B. crassifolia* have also been documented among rural Popoluca indigenous communities [25]. Oral preparations of bark are frequently used against diarrhea, while infusions, washes, poultices, or dry stem bark powder are employed for wound treatment. The bark is also used to treat vaginal infections and uterine inflammation.

Investigations conducted in the Makuxi indigenous community further documented the traditional use of *B. crassifolia*, mainly for the treatment of diarrhea, cuts, and inflammation [9]. Among the Ingarikó indigenous people, a bark decoction is traditionally used to treat stomach pain and diarrhea, whereas the Makuxi report the use of bark against intestinal worms and umbilical bleeding in newborns [28]. Its use against snakebites has also been documented in several indigenous communities [31]. Additionally, the traditional use of bark infusion for diabetes has been reported [32].

### 4.3 Medicinal Uses of Leaves, Flowers, and Fruits

Although less frequently cited, leaves, flowers, and fruits also have medicinal uses. Two reviews on medicinal plants from Northeastern Brazil record the use of leaves and flowers to treat pharyngitis and influenza [29], and leaf infusions against cough, diarrhea, dermatitis, and snakebite [26]. Fruit consumption has been reported for the traditional management of diabetes [32]. However, some ethnobotanical studies lack detailed information on the plant parts used and preparation methods. For example, the use of the species by Kayapó women to regulate menstrual flow has been reported without specification of the plant part used [30].

### 4.4 Nonmedicinal Uses and Socioeconomic Importance

Rural and Indigenous communities use *B. crassifolia* resources in various ways. Stem bark is used to obtain dyes, while the stem provides firewood and charcoal for cooking [9]. Additionally, the species is used for living fences, handicrafts, and carpentry, and has ornamental value [7, 33].

The fruits of *B. crassifolia* are appreciated for their characteristic aroma and flavor, serving as food resources for rural communities. Consumption occurs both in natura and in the preparation of culinary products. In large urban centers, the commercialization of frozen fruit pulp has expanded its availability for the preparation of sweets, cookies, cakes, crystallized fruits, ice creams, sorbets, gelatins, juices, liqueurs, jams, nectars, and other products [17, 33].

The economic and nutritional value of *B. crassifolia* is also associated with the nutritional and functional characteristics of its fruits [7]. Moreover, planned cultivation can contribute to supporting pollinators and restoring degraded areas [34, 35]. These actions may contribute to the conservation of *B. crassifolia* and the sustainable exploitation of its resources, ensuring socioeconomic and environmental benefits. The traditional uses of *B. crassifolia* have stimulated phytochemical and pharmacological investigations, progressively revealing the chemical composition of the species.

## 5 Phytochemistry

**FIGURE 2:**
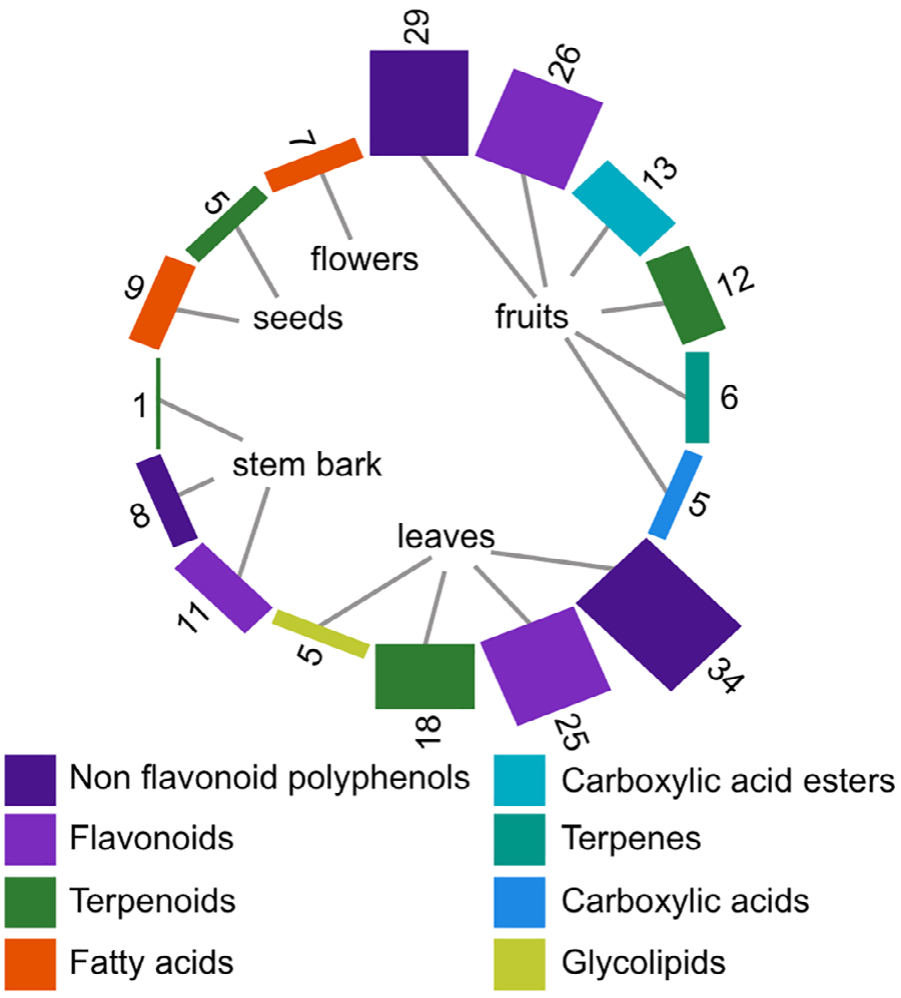
Distribution of phytochemicals reported in *Byrsonima crassifolia* (L.) Kunth according to plant part and chemical class. Numbers indicate the number of reported compounds.

The Malpighiaceae family harbors nearly 1,500 species; however, only a few have been investigated regarding their phytochemistry and pharmacological activities [12, 13, 36]. Studies on the chemical constitution of *B. crassifolia* reveal a predominance of polyphenolic compounds, with nonflavonoid polyphenols and flavonoids as the most representative classes, predominantly found in leaves, stem bark, and fruits (Figure 2 and Table S1), and sharing a recurrent phytochemical profile within the family [12, 13].

### 5.1 Nonflavonoid Polyphenols

**FIGURE 3:**
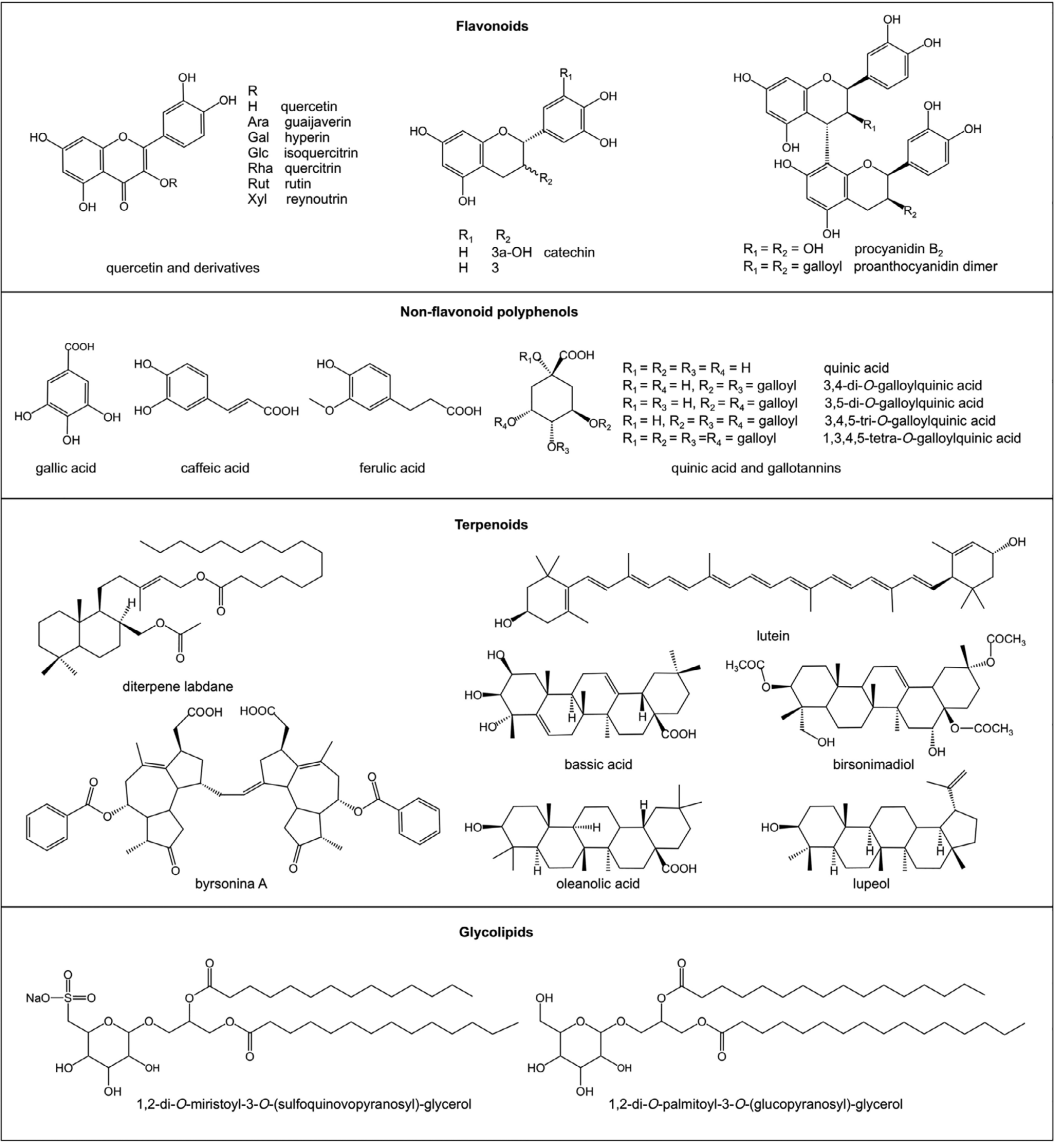
Representative phytochemicals reported in *Byrsonima crassifolia* (L.) Kunth.

Non‐flavonoid polyphenols constitute the largest group of secondary metabolites in *B. crassifolia* (71 compounds), mainly comprising gallotannins and phenolic acids (Figure 3). Gallotannins represent the main fraction (52 compounds), varying in complexity (from mono‐ to penta‐galloyl) and glycosylation (with pentosides and hexosides) [37, 38, 39, 40], followed by various phenolic acids, including gallic, caffeic, ferulic, p‐coumaric, and protocatechuic acids [41, 42, 43]. Phenolic esters, such as ethyl, methyl, and n‐butyl gallates, have been identified in leaves and fruits [42, 44, 45]. In contrast, simple phenols (e.g., pyrogallol) and ellagitannins (ellagic acid galloyl hexoside) have been reported only in specific tissues [40, 45].

### 5.2 Flavonoids

*B. crassifolia* also contains a large number of flavonoids (62 compounds), mainly flavonols, proanthocyanidins, and monomeric flavan‐3‐ols in its aerial parts, showing a distribution similar to that of gallotannins. Among flavonols, glycosylated and galloylated derivatives of quercetin and glycosylated isorhamnetin show the greatest structural variety in foliar tissues and fruits, supplemented by kaempferol [40, 44, 45, 46, 47]. Dimeric and trimeric proanthocyanidins esterified with galloyl groups occur in fruits and stem bark, respectively [38, 48]. Although represented by fewer compounds, monomeric flavan‐3‐ols are widely distributed across different plant organs [39, 49]. Additionally, anthocyanidins (cyanidin), flavanonols (taxifolin), flavones (apigenin and luteolin), and flavanones (hesperidin) have been less frequently reported [40, 42, 50, 51].

### 5.3 Terpenes and Terpenoids

The terpenoid profile of *B. crassifolia* is complex and broad (42 compounds); however, the greatest diversity has been identified in its leaves. Triterpenoids (C_30_) are the dominant class, with an abundant variety of pentacyclic carbon skeletons from the lupane series (lupeol, lupeanolic acid, betulin, and betulinaldehyde), oleanane (oleanolic acid, and β‐amyrins), and ursane (ursenaldehyde, α‐amyrins, and betulinic acid) [44, 46, 52, 53, 54]. C_29_ triterpenoids, such as β‐sitosterol and its glycoside (daucosterol), belonging to the steroidal triterpenoid class (phytosterols), have been detected only in leaves [44, 55]. In fruits, the typical yellow pigmentation of pericarp and mesocarp is mainly due to xanthophylls (C_40_ carotenoids) [56], while their seeds contain less frequently reported terpenoids, including labdane‐type diterpenoids (C_20_), the dimeric guaianolide sesquiterpene lactones birsoninas A and B (C_48_ and C_44_, respectively), and birsonimadiol (an oleanane‐type triterpenoid) [57, 58, 59].

### 5.4 Other Organic Compounds

Numerous volatile metabolites identified in *B. crassifolia* fruits, such as carboxylic acids and their esters, are the main determinants of its fruity and rancid cheese‐like aroma [42, 60, 61]. Complementarily, a mixture of fixed carboxylic acids (stearic, lauric, linoleic, myristic, oleic, palmitic, and palmitoleic) and volatile carboxylic acids (hexanoic and butyric) coexists in seed oil, whose rancid cheese‐like aroma, also present in fruits, is attributed to the volatile fraction [60]. Similarly, saturated fatty acids (C_14_–C_18_, e.g., myristic, palmitic, and stearic) and unsaturated fatty acids (C_18_–C_22_, e.g., oleic, linoleic, and eicosadienoic), mainly as monoglycerides, constitute flower oil [62]. Additionally, two classes of glycerolipids, sulfoquinovosyl diacylglycerols (SQDGs) and glycosyl diacylglycerols, have so far been identified only in leaves [44, 46, 63].

Overall, leaves and fruits are the most extensively characterized organs, particularly regarding polyphenolic compounds, whereas stem bark, seeds, and flowers exhibit comparatively less diverse profiles. However, these differences may reflect variations in analytical approaches and the extent to which each organ has been investigated, rather than their actual phytochemical complexity. The documented phytochemical diversity of *B. crassifolia* provides a basis for examining the relationship between its chemical composition and the reported biological activities.

## 6 Pharmacological Activities

**FIGURE 4:**
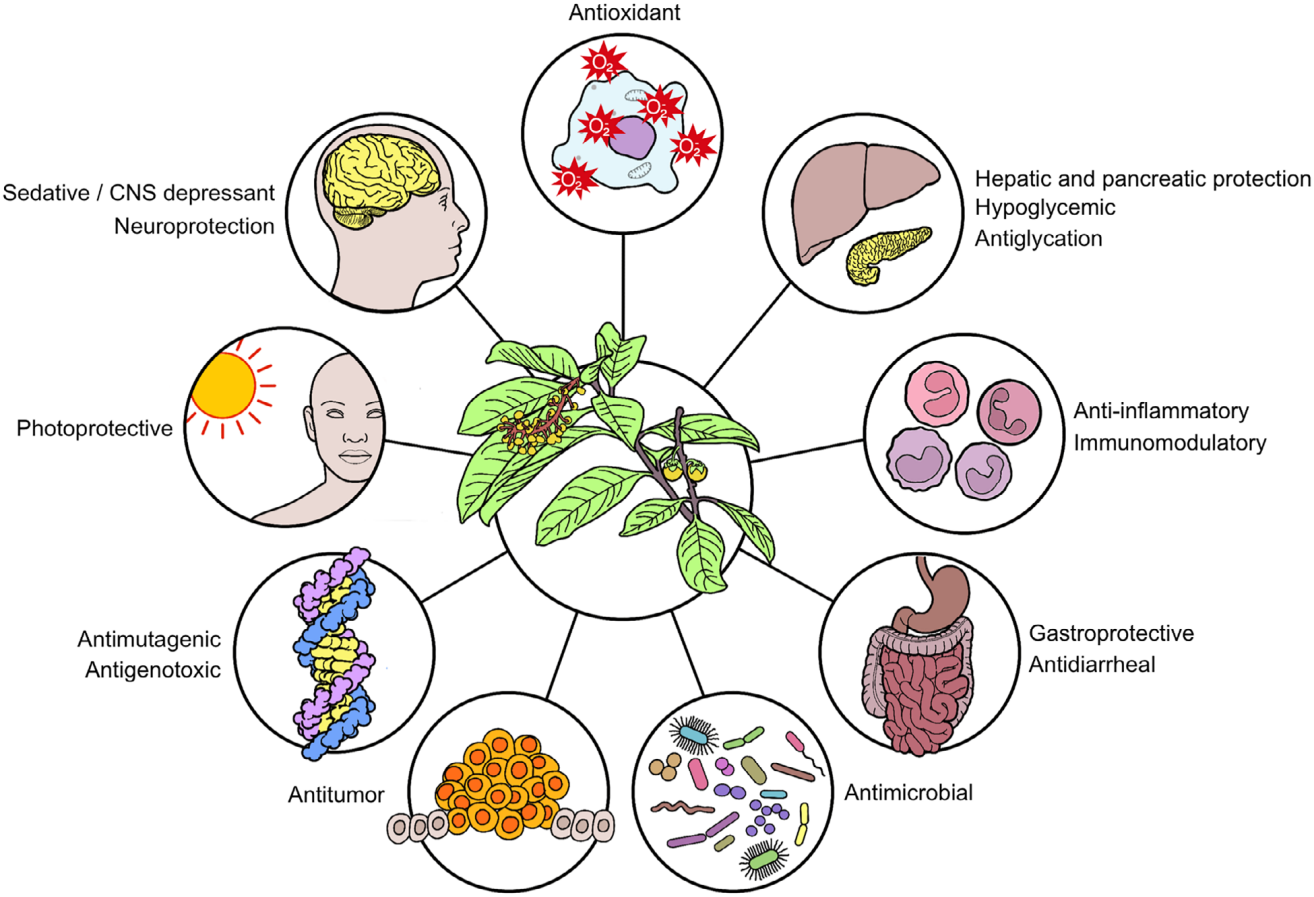
Pharmacological activities reported for *Byrsonima crassifolia* (L.) Kunth.

*B. crassifolia* exhibits a broad spectrum of reported biological activities, including antioxidant, antimicrobial, anti‐inflammatory, gastroprotective, neuromodulatory, metabolic, photoprotective, chemopreventive, and antitumor effects, partially supporting its diverse ethnomedicinal uses (Table 3 and Figure 4).

**TABLE 3:** Pharmacological properties of *Byrsonima crassifolia* (L.) Kunth.

Activity	Plant part	Extract	Major phytochemicals	Models/organisms	Key findings	References
Antimicrobial	Leaves	80% methanolic	simple phenolics, galloylquinic acid derivatives, flavonoids	MIC; *Cryptococcus* spp.; hemolysis and toxicity (*Galleria mellonella*)	MIC = 1–16 µg/mL; no hemolysis or toxicity	[45]^a^
	Leaves	Methanolic	—	Disc diffusion*; Klebsiella pneumoniae*	Synergism with imipenem (11 mm), piperacillin/tazobactam (2 mm), polymyxin B (26 mm)	[64]
	Leaves	Chloroform	triterpenes and hydrocarbons	MIC; *Mycobacterium tuberculosis*	MICs 2.5 µg/mL (bassic acid), 31.25 µg/mL (triterpenic mixtures)	[53]^a^
	Seeds	Hexane	Diterpenes	MIC, MBC*; Bacillus subtilis, Salmonella paratyphi, Salmonella typhi*, and *Pseudomonas aeruginosa*	MIC 18.79–41.28 µg/mL; MBC 250–500 µg/mL	[57]
	Leaves	Crude extract (ethyl acetate, methanol and methanol:water 1:1)	Phenolic acids, flavonoids	MIC; *Salmonella Typhimurium, Escherichia coli, Staphylococcus aureus, Enterococcus faecalis*	MICs 12.5 µg/mL (Gram‐negative), 125 µg/mL (Gram‐positive)	[41]
	Leaves	Hexane, 95% ethanolic and aqueous	Triterpenoid sapogenins, flavonoids	Growth inhibition; *Trypanosoma cruzi* (tripomastigote); toxicity (*Artemia salina*)	IC_90_ ≈ 89–99 µg/mL; no toxicity	[54]
	Bark	Methanolic	—	Disc diffusion; *Leishmania mexicana* (promastigote)	IC_50_ = 14 µg/mL	[65]
	Bark, root	Hexane, ethyl acetate and methanol	Flavonoids, saponins, glycosides, tannins, terpenes/sterols	Disc diffusion; *Micrococcus luteus*, *Shigella flexneri, Staphylococcus epidermidis, Streptococcus pneumoniae, K. pneumoniae, P. aeruginosa, S. typhi, S. aureus*	Inhibition zones 17–26 mm	[66]
	Root	Aqueous (10% and 20%)	—	Disc diffusion*; K. pneumoniae, S. aureus, P. aeruginosa*	Inhibition zones 8.6 mm, 12.4 mm, 8.2 mm	[67]
Anti‐inflammatory and Immunomodulatory	Seeds	Hexane and fractions	Triterpene	Paw edema (carrageenan, formaldehyde, histamine), ear edema, arthritis, granuloma in mice; LPS‐stimulated macrophages	↓Paw/ear edema (↓MPO), ↓arthritis, ↓granuloma; ↓NO, ↓PGE_2_, ↓COX‐2, ↓IFN‐γ, ↓TNF‐α, ↓IL‐6 (macrophages)	[58]
	Seeds	Hexane, chloroform and methanol	—	Paw edema (carrageenan, formaldehyde, histamine), ear edema, granuloma, NO modulation in rats/mice	↓Paw/ear edema, ↓granuloma, ↓NO	[68]
	Bark	Petroleum ether, chloroform and methanol	—	Ear dermatitis (croton oil) in male mice	↓Dermatitis	[69]
	Bark	Acetone	—	Human whole blood + *S. aureus*	↓*S. aureus* load (indirect effect)	[70]
	Pulp	Aqueous	carotenoids	Healthy female rats supplemented (14 days)	↑Antioxidant capacity, ↓carbonylated proteins, ↓lipid peroxidation (TBARS) (plasma); ↓TNF‐α (liver, kidneys)	[71]
Wound healing	Seeds	Hexane	—	Wound healing in diabetic male rats	↑Lesion contraction rate, ↑granulation tissue, ↑hydroxyproline, ↑total protein, ↑DNA, ↑SOD, ↑CAT	[68]
Neuromodulatory	Bark	Dichloromethane and 80% ethanolic	Alkaloids, phenolic compounds, saponins, tannins, triterpenes	Motor activity, exploratory behavior (hole‐board), rectal temperature, motor coordination, pentobarbital‐induced sleep, hot plate test in mice	↓Motor activity and coordination, ↓curiosity, ↑sleep time, hypothermia, mild analgesia	[43]
	Bark, leaves	2% Acetic acid, ethanolic	—	Hippocratic screening in rats	↓Motor activity, analgesia, back tonus, enophthalmos, palpebral ptosis, ear blanching, positive Robichaud, catalepsy and hypothermia	[72]
	Bark, leaves	Aqueous	—	Motor activity, exploratory behavior (hole‐board), rectal temperature, pentobarbital‐induced sleep in mice	↓Motor activity and exploratory behavior, ↓rectal temperature; ↑sleep time	[73]
	Aerial parts	Hexane and methanol	Flavonoids	Elevated plus maze (EPM), forced swimming test (FST), open field, induced seizures, pentobarbital‐induced sleep	↓Immobility time in FST; no anxiolytic, sedative, or anticonvulsant effects; no locomotor impairment	[50]
	Fruits	Acetone	Ascorbic and gallic acids, carotenoids and total phenolics, quercetin	Supplementation in adult and aged rats (15 days)	↓Brain oxidative stress (aging), ↑GSH/GSSG, ↓lipid peroxidation (MDA)	[74]
	Fruits	Acetone	Ascorbic and gallic acids, carotenoids and total phenols, quercetin	Supplementation in septic rats (15 days)	↓Brain oxidative stress (sepsis), ↑SOD, ↓lipid peroxidation (MDA), ↓GSH/GSSG	[75]
Metabolic	Seeds	Hexane	Sesquiterpene lactones	Diabetic mice; oxidative stress in β cells (RIN‐5F)	↓Glycemia, ↓TC, ↓LDL, ↓TG, ↑HDL; ↑insulin (serum, pancreas); ↑SOD, ↑CAT, ↑GSH, ↑GPx (liver, kidneys, pancreas); ↓TNF‐α; ↓ROS in β cells	[59]
	Fruits, seeds	Hexane, chloroform and methanol	—	Diabetic rats	↑SOD, ↑GSH, ↑GSSG; ↑CAT, ↑GK, ↓G6Pase, ↑glycogen (liver); ↑insulin (plasma); ↓lipid peroxidation (TBARS); ↓AGEs	[76]
	Seeds	Hexane, chloroform, methanol and aqueous	—	Diabetic rats; oxidative stress in β cells (RIN‐5F)	↓Glycemia, ↓TC, ↓TG, ↑HDL; ↑SOD, ↑CAT, ↑GSH, ↑GPx (pancreas); ↓lipid peroxidation (TBARS); ↑insulin (serum, pancreas); ↓ROS in β cells	[77]
	Pulp	Beverage	—	Female rats on a high‐fat diet (60 days)	↓TC, ↓ALT, ↓IL‐1β, ↑antioxidant capacity (plasma/liver), prevention of ↑hepatic TNF‐α and apoptosis	[78]
	Pulp	Aqueous	—	Healthy rats supplemented (30 days)	↓Food intake, ↓body fat, ↓lipid peroxidation (TBARS); ↑antioxidant capacity, ↑CAT (plasma)	[79]
	Pulp	95% Ethanolic	Citric acid, flavonoids and non‐flavonoid polyphenols, VOCs	Antiglycation activity; enzyme inhibition; viability of MRC‐5 fibroblasts	↑Antioxidant activity; ↓AGEs, ↓α‐glucosidase; no cytotoxicity	[42]
Gastroprotective	Leaves	Methanolic	Phenolic acids, flavonoids	Gastric ulcer, diarrhea, intestinal motility in rats/mice; NO (macrophages); Ames test in *S. typhimurium*; antibacterial activity	↓Ulcers (ethanol, ethanol‐HCl); healing (acetic acid ulcer); ↑mucus; ↓diarrhea and intestinal motility; NO modulation (mucosa, macrophages); inhibition of *E. coli, S. aureus*, and *H. pylori*; no mutagenicity	[47]^a^
	Leaves	65% Ethanolic	Total phenolics	Gastric ulcer in rats	↓Ethanol‐induced ulcer; ↑antioxidant activity	[80]
	Leaves	Methanolic	Triterpenes, sterols, flavonoids, aromatic ester, amino acids	spasmogenesis and serotonin antagonism in isolated rat gastric fundus	Spasmogenic effects and noncompetitive 5‐HT antagonism	[44]
	Bark, leaves	2% Acetic acid (leaves), ethanolic (bark)	—	Pharmacological screening in isolated rat gastric fundus, duodenum and ileum	Spasmogenic effects (gastric fundus) and biphasic effects (duodenum/ileum)	[72]
Photoprotective	Leaves	45% Ethanolic	Gallic acid, flavonoids	UVB‐irradiated HaCaT cells; antioxidant activity in porcine skin (*ex vivo*)	↓Lipid peroxidation (MDA); ↓TNF‐α, ↓IL‐6, ↓NF‐κB; ↑skin antioxidant activity	[49]
	Leaves	45% Ethanolic		L929 fibroblasts and UVB‐irradiated mice	Preserves GSH (fibroblasts); preserves GSH, ↓MMP‐9, ↓MPO; ↓IL‐1β, ↓IL‐6 (mouse skin)	[81]
Chemopreventive	Leaves	70% Ethanolic	—	Ames test in *Salmonella typhimurium*	No mutagenicity and antimutagenicity	[82]^a^
	Leaves	70% Ethanolic	—	Cytotoxicity, cell proliferation, and cell cycle in HepG2 and GAS cells	Low cytotoxicity; no effect on cell proliferation at non‐cytotoxic concentrations; modulation of cell‐cycle distribution	[83]^a^
	Pulp	Oil and ethanolic	Lutein, total phenolics, fatty acids, triglycerides	Cytotoxicity and cytoprotection against H_2_O_2_ in HepG2 cells	↑Antioxidant capacity (ethanolic extract); ↑cytoprotection against H_2_O_2_ (oil); no cytotoxicity; potential antithrombogenic, antihypercholesterolemic, and anti‐atherosclerogenic activities	[84]
	Leaves	70% Ethanolic	Galloylquinic acid derivatives, flavonoids	Apoptosis/necrosis, micronucleus, comet assay, ROS and RT‐qPCR in HepG2 and MNP01 cells	No apoptosis, genotoxicity, or mutagenicity; antimutagenic/antigenotoxic effects; ↓H_2_O_2_‐induced oxidative stress (MNP01); modulation of antioxidant and xenobiotic‐metabolism genes	[85]
Antitumor	Leaves	Hydroalcoholic	—	Yoshida tumor in rodents	26% Inhibition	[86]
	Pulp	Aqueous	Carotenoids, total phenolics	MTT, cell cycle and apoptosis in ovarian cancer cells A2780 and ACRP (cisplatin‐resistant)	↑Antioxidant activity; ↓cell viability; G0/G1 cell cycle modulation/arrest; ↑apoptosis	[87]
	Pulp	Aqueous and 80% ethanolic	Carotenoids, phenolic compounds	WST in breast cancer cells MCF‐7 and MDA‐MB‐231 (invasive)	↑Antioxidant capacity; ↑antiproliferative activity	[88]
	Pulp	Aqueous and ethanolic	—	MTT, cell cycle and apoptosis in human prostate cancer cells PC‐3	↑Antiproliferative effect; cell cycle modulation/arrest; ↑Apoptosis	[89]
	Pulp	60% Hydroethanolic	Quercetin, phenolic acids	MTT, migration (scratch/wound‐healing) in skin fibroblasts (CCD‐1059 Sk) and human cutaneous melanoma cells (SK‐Mel‐28)	↑Viability (CCD‐1059 Sk cells); no inhibition of Sk‐Mel‐28 cells	[90]

CAT, catalase; CT, total cholesterol; COX‐2, cyclooxygenase‐2; DNA, deoxyribonucleic acid; G6Pase, glucose‐6‐phosphatase; GAS, primary normal gastric epithelium; GK, glucokinase; GPx, glutathione peroxidase; GSH, reduced glutathione; GSSG, oxidized glutathione; HDL, high‐density lipoprotein; H_2_O_2_, hydrogen peroxide; IC_50_, half maximal inhibitory concentration; IC_90_, 90% inhibitory concentration; IFN‐γ, interferon gamma; IL‐1β, interleukin 1 beta; IL‐6, interleukin 6; LDL, low‐density lipoprotein; MBC, minimum bactericidal concentration; MDA, malondialdehyde; MIC, minimum inhibitory concentration; MMP‐9, matrix metalloproteinase 9; MPO, myeloperoxidase; MTT, 3‐(4,5‐dimethylthiazol‐2‐yl)‐2,5‐diphenyl tetrazolium bromide; NO, nitric oxide; PGE_2_, prostaglandin E_2_; ROS, reactive oxygen species; RT‐qPCR, reverse transcription quantitative polymerase chain reaction; SOD, superoxide dismutase; TBARS, thiobarbituric acid reactive substances; TG, total triglycerides; TNF‐α, tumor necrosis factor alpha; UVB, ultraviolet B radiation; VOC, volatile organic compound; WST, water‐soluble tetrazolium.

^a^ Plant material reported as *Byrsonima fagifolia* Nied. in the original study, a taxonomic synonym of *Byrsonima crassifolia* (L.) Kunth.

### 6.1 Antioxidant Activity

The antioxidant potential of *B. crassifolia* is well documented, similar to that of other species in the family [13], and is frequently associated with its polyphenols and xanthophylls, which contribute to its antioxidant capacity [84, 91, 92], as demonstrated by complementary antioxidant assays such as ABTS, DPPH, FRAP, and ORAC [93]. These chemical assays indicate antioxidant activity through radical scavenging and reducing capacity, although the magnitude of the response varies according to the assay and extract composition. Specifically, extracts and fractions have shown radical‐scavenging activity in aqueous systems [40, 84, 87, 88], as well as inhibition of oxidation in lipid‐based models [91, 94]. Cell‐based studies demonstrated attenuation of oxidative damage in erythrocytes and intracellular oxidative stress in pancreatic *β*‐cells [40, 59]. In vivo, extracts and isolated compounds have reduced lipid peroxidation and modulated endogenous antioxidant defenses; however, these responses are not uniform across experimental preparations, models, or doses [59, 71, 74, 75, 76, 77, 79, 81].

### 6.2 Antimicrobial Activity

The antimicrobial activity of *B. crassifolia* is supported by studies employing both isolated metabolites and organic extracts in vitro. Leaf extracts reported under the synonym *B. fagifolia* Nied. have shown antifungal activity against clinical isolates of *Cryptococcus neoformans* and *C. gattii*. This activity was associated with galloylquinic acid derivatives, the major constituents of the extract, particularly 3,4,5‐tri‐O‐galloylquinic acid and 1,3,4,5‐tetra‐O‐galloylquinic acid, which exhibited inhibitory activity comparable to that of the extract and fluconazole [45]. Methanolic leaf extracts also potentiated the activity of imipenem, piperacillin/tazobactam, and polymyxin B against carbapenemase‐producing *Klebsiella pneumoniae*, although the extract was not chemically characterized [64]. In addition, nonpolar constituents isolated from leaf material reported as *B. fagifolia* Nied., including the triterpenes betulinic acid, lupeol, α‐ and β‐amyrin (and their acetates), as well as the alkane dotriacontane, exhibited activity against *Mycobacterium tuberculosis*, with betulinic acid displaying potency comparable to ethambutol [53].

Diterpenes isolated from *B. crassifolia* seeds have shown antimicrobial activity, particularly against Gram‐negative bacteria, as demonstrated for labda‐17‐acetoxy‐13E‐en‐15‐palmitate [57]. In another study, phenolics – gallic and ferulic acids and quercetin – detected in leaves showed antibacterial activity when tested individually. However, among the fractions evaluated, the methanolic fraction showed both the highest phenolic content and the strongest antibacterial activity, supporting an association between phenolic content and the observed effects [41].

Ethanolic fractions containing triterpene sapogenins (oleanolic, lupeanolic acids and their derivatives) and flavonoids (catechin, epicatechin, and quercetin derivatives) inhibited *Trypanosoma cruzi* trypomastigotes [54]. In addition, methanolic bark extract exhibited leishmanicidal activity against *Leishmania mexicana* promastigotes [65]. Root extracts containing flavonoids, saponins, and glycosides also showed antibacterial activity against both Gram‐positive and Gram‐negative bacteria [66]. Overall, antimicrobial evidence for *B. crassifolia remains* predominantly based on in vitro studies, without validation in experimental infection models.

### 6.3 Anti‐Inflammatory and Immunomodulatory Activity

Preclinical studies have demonstrated anti‐inflammatory activity of *B. crassifolia* in both in vivo and in vitro models. Birsonimadiol, a triterpenoid isolated from the hexane seed extract, exhibited consistent activity in several acute and chronic inflammatory models, with effects comparable to those of indomethacin and diclofenac in the respective models. In murine macrophages, birsonimadiol also reduced the production of inflammatory mediators, including NO, PGE_2_, and pro‐inflammatory cytokines [58]. Similarly, anti‐inflammatory effects were previously demonstrated for the hexane seed extract in acute and chronic models, with efficacy similar to that of indomethacin and dexamethasone, and these effects were associated, at least in part, with inhibition of iNOS activity and modulation of NO production [95].

Extracts from other plant organs also exhibit anti‐inflammatory potential. Chloroform bark extract reduced croton oil‐induced ear dermatitis in vivo, with activity comparable to that of indomethacin and possibly related to terpenes, sterols, and glycerolipids [69]. In addition, acetone bark extract potentiated the antimicrobial activity of human leukocytes against Staphylococcus aureus in vitro, although it showed no direct microbicidal activity [70]. More recently, pulp extract supplementation in healthy female rats improved plasmatic oxidative status and reduced tissue TNF‐α levels. The extract contained lutein and phenolic compounds, whose presence was associated with these effects [71]. For seeds, anti‐inflammatory evidence includes both extracts and isolated birsonimadiol in animal and cellular models, whereas the anti‐inflammatory and immunomodulatory effects reported for bark and fruit remain primarily associated with chemically complex extracts.

### 6.4 Neuromodulatory Activity

Preclinical studies have predominantly reported depressant effects of *B. crassifolia* on the central nervous system. Mice treated with a phytochemically characterized hydroalcoholic bark extract showed neurodepressant effects, including reduced motor and exploratory activity and potentiation of pentobarbital‐induced sleep, suggesting modulation of central neurotransmission [43]. These findings are consistent with earlier reports of central depressant effects in rodents exposed to crude bark and leaf extracts with limited phytochemical characterization [72, 73].

In contrast, a flavonoid‐standardized extract produced antidepressant‐like effects in mice, reducing immobility time without affecting locomotor activity. This response was suggested to involve flavonoids such as rutin, hesperidin, and quercetin [50]. The central effects of *B. crassifolia* extend to pathophysiological models. Oral supplementation with fruit extract modulated nerve excitability and oxidative stress markers in aging and septic models. These effects were associated with the anti‐inflammatory and antioxidant activities of lutein and quercetin [74, 75]. The central effects observed are contrasting, with neurodepressant activity reported mainly for crude or less chemically characterized extracts, whereas antidepressant‐like and neuroprotective effects have been observed with phytochemically characterized preparations.

### 6.5 Metabolic Activity

Various in vivo studies demonstrate that *B. crassifolia* modulates metabolic pathways associated with glucose and lipid homeostasis. Byrsoninas A and B, two dimeric guaianolide sesquiterpene lactones isolated from seeds, exhibited pronounced hypoglycemic activity in diabetic mice, accompanied by improvements in lipid metabolism, insulin status, and antioxidant defenses. Both compounds also protected pancreatic β‐cells against AGE‐induced oxidative stress in vitro [59]. Consistent with these findings, oral supplementation with seed extracts improved glycemic and lipid homeostasis, insulin status, and antioxidant defenses in diabetic rats [76, 77].

Fruit‐derived preparations also exhibit metabolic effects. Supplementation with a fruit‐based beverage normalized total cholesterol and exerted hepatoprotective effects in rats fed a high‐fat diet, attenuating oxidative and inflammatory alterations [78]. In healthy rats, supplementation with fruit pulp extract reduced food intake and body fat and increased plasma antioxidant capacity [79]. In addition, phenolic acid‐ and flavonoid‐rich fruit extract inhibited α‐glucosidase activity in vitro, with potency comparable to acarbose, and reduced AGEs formation [42]. Antidiabetic evidence is strongest for seed extracts and isolated byrsoninas in diabetic models, whereas evidence for the metabolic effects of fruit‐derived preparations derives from more heterogeneous experimental models.

### 6.6 Gastroprotective Activity

A leaf extract from plant material reported as *B. fagifolia* Nied. was evaluated in murine gastric ulcer models. It reduced acute ethanol‐ and ethanol/HCl‐induced gastric lesions and promoted healing of acetic acid‐induced ulcers by stimulating proliferative factors and increasing mucus production. The extract also showed in vitro action against *H. pylori*. These effects were associated with phenolic compounds, including galloylated and glycosylated flavonoids [47]. Further gastroprotective evidence was obtained in an ethanol‐induced in vivo ulcer model, in which higher doses of a hydroalcoholic extract reduced ulcerative lesions, an effect associated with the antioxidant capacity of its phenolic compounds [80].

Previous pharmacological evidence showed a more complex gastrointestinal response. Bioactivity‐guided fractionation of a methanolic leaf extract identified flavonoids, triterpenes, and non‐protein amino acids with spasmogenic and serotonin‐antagonistic properties in isolated rat gastric fundus [44], extending earlier findings with bark and leaf extracts that showed spasmogenic and biphasic responses in isolated gastrointestinal tissues [72]. Gastroprotective effects observed in experimental ulcer models partially support the traditional use of *B. crassifolia* for gastrointestinal disorders, whereas activity against *H. pylori* remains limited to in vitro evidence.

### 6.7 Photoprotective Activity

A purified leaf extract and flavonoid‐enriched fraction of *B. crassifolia* were investigated against UVB‐induced photodamage. Both preparations showed similar photoprotective effects in UVB‐irradiated keratinocytes, attenuating oxidative and inflammatory responses. Additionally, topical formulations of both preparations also increased antioxidant activity in ex vivo swine skin, while the enriched fraction showed greater cutaneous retention of catechin. These photoprotective effects were attributed to the synergistic anti‐inflammatory and antioxidant actions of polyphenols [49]. A complementary investigation compared the same enriched fraction with isolated reference compounds (catechin, epigallocatechin gallate, and isoquercitrin) [81]. The fraction preserved GSH in irradiated fibroblasts more effectively than the isolated compounds at the same concentration. In irradiated hairless mice, topical application of a 1% gel formulation promoted phenolic retention in the epidermis and dermis and attenuated oxidative and inflammatory alterations. Notably, only the enriched fraction inhibited UVB‐induced metalloproteinase‐9. Although the enriched fraction showed advantages over the isolated reference compounds for some photoprotective effects, the relative contribution of individual constituents and their interactions remains unclear. Current evidence is preclinical, without validation in humans.

### 6.8 Chemopreventive Activity

*B. crassifolia* preparations have shown chemopreventive potential against mutations and oxidative DNA damage induced by mutagenic or carcinogenic agents. Gastric (MNP01) and hepatic (HepG2) cells were treated with a hydroethanolic leaf extract containing galloylquinic acid derivatives and quercetin. The extract exhibited antimutagenic and antioxidant effects in MNP01 cells and protected HepG2 cells against benzo[a]pyrene‐induced genotoxic and mutagenic damage. Gene‐expression analyses suggested that these responses may involve modulation of antioxidant defenses and xenobiotic metabolism [85]. Similarly, a leaf extract from plant material reported as *B. fagifolia* Nied was nonmutagenic and showed moderate to strong antimutagenic activity against different mutagens in the Ames test [82]. Sequential supercritical extraction of lyophilized pulp yielded an ethanolic extract containing lutein, phenolics, and flavonoids and an oil rich in unsaturated fatty acids and lutein. Despite the higher antioxidant activity of the extract, only the oil protected HepG2 cells against hydrogen peroxide‐induced damage [84]. Complementary evidence of a *B. fagifolia* Nied. leaf extract in human gastric and hepatic cells indicated low cytotoxicity and no effect on cell proliferation at non‐cytotoxic concentrations, although changes in cell‐cycle were observed [83]. Available evidence of chemopreventive activity is restricted to in vitro models, and the contribution of individual constituents to these effects remains unclear.

### 6.9 Antitumor Activity

Few studies have evaluated the antitumor potential of *B. crassifolia* preparations in experimental tumor models. An aqueous pulp extract containing phenolic compounds and carotenoids reduced the viability of ovarian cancer cells (A2780) and their cisplatin‐resistant variant (ACRP), with cell‐cycle alterations in both cell lines but a more pronounced apoptotic response in A2780 cells [87]. Aqueous and ethanolic pulp extracts also showed dose‐dependent antiproliferative effects against non‐invasive (MCF‐7) and invasive (MDA‐MB‐231) breast cancer cells, with greater activity generally observed for the ethanolic extract, particularly against MCF‐7 cells; this extract also exhibited superior antioxidant capacity and greater abundance of phenolic compounds [88]. Similarly, aqueous and ethanolic pulp extracts inhibited PC‐3 prostate cancer cell growth, although their effects on cell‐cycle distribution and apoptosis differed between preparations [89]. In contrast, a hydroethanolic pulp extract and isolated phenolic compounds did not significantly reduce the viability of human cutaneous melanoma SK‑MEL‑28 cells in vitro. Furthermore, syringic acid, the main phenolic compound in the extract, did not inhibit SK‑MEL‑28 cell migration at the concentrations tested [90]. An earlier in vivo screening with a hydroalcoholic leaf extract showed only 26% inhibition of Yoshida tumor growth, below the >40% threshold used to define antitumor activity [86]. Overall, antitumor evidence is predominantly in vitro and varies according to the preparation and cancer cell model; in vivo evidence has not demonstrated substantial tumor inhibition.

## 7 Toxicity

Limited toxicological data exist for *B. crassifolia* extracts in rodents (Table 4). A comprehensive safety assessment of natural products requires acute and repeated‐exposure studies covering multiple toxicological endpoints [96]. An oral dose of 1,000 mg/kg of leaf extract showed no acute clinical toxicity, mutagenicity, or genotoxicity. However, renal apoptosis was observed at this dose after 72 h [64]. Oral administration of an aerial‐parts extract at 2,000 mg/kg produced neuromotor alterations and constipation over 14 days [50], whereas a single oral dose of seed extract up to 3,000 mg/kg produced no apparent toxicity or mortality during a 72‐h observation period [76]. Similarly, a leaf extract administered orally at 500 mg/kg showed no evidence of hepatic or renal toxicity or mutagenicity over 14 days [47]. In contrast, an early intraperitoneal toxicological study of stem bark and leaf extracts reported signs consistent with neurodepression and autonomic alterations [72]. Available data suggest apparent safety following oral administration in preclinical studies; however, differences in extracts, doses, observation periods, and toxicological endpoints limit comparisons. To date, clinical safety in humans has not been evaluated.

**TABLE 4:** Acute toxicity evaluation of *Byrsonima crassifolia* (L.) Kunth extracts.

Extract (plant part)	n	Doses (mg/kg)	Monitoring	Signs	Body weight	Geno‐toxicity	Muta‐genicity	Histopathology / Necropsy	Mortality	References
Methanolic (leaves)	10	10, 100, 1,000 (oral)	72 h	A	NL	A	A	renal apoptosis (1,000 mg/kg)	0	[64]
Methanolic (aerial parts)	3	500, 2,000 (oral)	24 h (14 days)	↓locomotor activity, ↓grip strength, constipation	NL	NA	NA	NA	0	[50]
Hexanic (fruits, seeds)	—	1,000, 2,000, 3,000 (oral)	72 h	A	NL	NA	NA	NA	0	[76]
Methanolic (leaves)	6	500 (oral)	14 days	A	NL	NA	A	NA	0	[47]^a^
ethanolic (bark)	—	31, 100, 310 (i.p.)	24 h	↓motor activity, ↓respiratory rate, analgesia, back tonus, enophthalmos, palpebral ptosis, ear blanching, catalepsy, hypothermia	↓7th day	NA	NA	7th day: atony, constipation, and gastrointestinal vasoconstriction; ↓muscle mass, liver; nodules (liver, spleen, testes); hemorrhagic lungs	0	[72]
2% acetic acid (leaves)	—	31, 100, 310 (i.p.)	24 h	↓motor activity, ↓respiratory rate, analgesia, back tonus, enophthalmos, palpebral ptosis, ear blanching, catalepsy, hypothermia	NL	NA	NA	7th day: possible liver reduction	< 24 h (310 mg/kg)	[72]

A, absence; NA, not assessed; NL, not lost

^a^ Plant material reported as *Byrsonima fagifolia* Nied. in the original study, a taxonomic synonym of *Byrsonima crassifolia* (L.) Kunth.

## 8 Future Perspectives

The available evidence highlights the phytotherapeutic potential of *B. crassifolia*; however, the strength and level of evidence vary considerably among biological activities. Although some isolated compounds have been pharmacologically investigated, most studies still employ non‐standardized extracts or lack comprehensive quantification of marker molecules. Synergistic interactions among constituents are frequently proposed to explain the activity of complex extracts and fractions, but these interactions have not been experimentally demonstrated. Additionally, subchronic and chronic toxicity data are scarce, and the absence of clinical investigations limits the translational potential of *B. crassifolia*. Future research should follow standardized guidelines for extract characterization, using complementary analytical methods, when possible, to identify and quantify chemical markers and improve reproducibility and comparability across studies [97]. Subsequent studies should investigate compound‐specific molecular targets and establish relationships between chemical composition and biological activity, while comprehensive preclinical toxicity assessment is needed to define safety profiles and support clinical investigation.

Finally, integration of ethnobotany, phytochemistry, pharmacology, and toxicology is essential [98] to advance the development of phytotherapeutic products from *B. crassifolia* and promote the sustainable valorization of Brazilian Cerrado biodiversity.

## 9 Conclusion

*Byrsonima crassifolia* represents a promising bioresource with diverse ethnomedicinal applications. Its stem bark, leaves, fruits, and seeds contain diverse phytochemicals, particularly non‐flavonoid polyphenols and flavonoids. *B. crassifolia* exhibits a broad range of pharmacological activities, although the strength of evidence varies across plant organs, preparations, and experimental models. Notably, seed‐derived terpenoids are among the isolated constituents with experimentally demonstrated antimicrobial, anti‐inflammatory, and hypoglycemic activities. Antioxidant properties frequently accompany these pharmacological effects, although their specific contribution to individual activities remains unclear. Current evidence, however, largely relies on non‐standardized extracts, with insufficient quantification of chemical markers, incomplete safety assessment, and no clinical validation.

This review integrates current knowledge on the ethnobotany, phytochemistry, pharmacology, and safety of *B. crassifolia*, and identifies key challenges that currently limit its phytotherapeutic development.

## Author Contributions

**João de Jesus Oliveira Junior**: conceptualization, investigation, writing – original draft, methodology, visualization, writing – review and editing. **Débora Luana Ribeiro Pessoa**: writing – review and editing, supervision, formal analysis. **Marilene Oliveira da Rocha Borges**: conceptualization, writing – review, editing, and supervision.

## Funding

This research received no specific grant from any funding agency in the public, commercial, or not‐for‐profit sectors.

## Conflicts of Interest

The authors declare no conflicts of interest.

## Supporting information




**Supporting File 1**: cbdv71756‐sup‐0001‐SuppMat.docx

## Data Availability

Data sharing is not applicable to this article as no new data were created or analyzed in this study.
