# Supplementary material for: Byrsonima crassifolia: Ethnobotany, Phytochemistry, and Pharmacology – An Update
Source: Chem Biodivers. 2026 Sep 27;23(9):e71756. doi: 10.1002/cbdv.71756 (PMC13616463; doi:10.1002/cbdv.71756)
Supplement: Supplementary file 1 [file CBDV-23-e71756-s001.docx]

**Suppementary information**

*Byrsonima crassifolia*: Ethnobotany, Phytochemistry, and Pharmacology – An Update

| **Table S1. Phytochemical profile of *Byrsonima crassifolia* (L.) Kunth** | | | |
| --- | --- | --- | --- |
| Leaves | | | |
| Class | Subclass | Identified compound | References |
| Flavonoids | Anthocyanidins | cyanidin | ^[1]^ |
|  | Flavonols | quercetin | ^[2–4]^ |
|  |  | quercetin 3-O-α-L-arabinopyranoside (guaijaverin) | ^[2,5]^ |
|  |  | quercetin 3-O-β-D-galactopyranoside (hyperin) | ^[2,6–8]^ |
|  |  | quercetin 3-O-β-D-glucopyranoside (isoquercetin) | ^[6,7,9,10]^ |
|  |  | quercetin 3-O-(6''-galloyl)-galactoside | ^[2]^ |
|  |  | quercetin-3-O-(2"-galloyl)-β-D-galactopiranoside | ^[9][a]^ |
|  |  | quercetin-3-O-(2"-galloyl)-β-D-glycopyranoside |  |
|  |  | quercetin-3-O-α-L-galactopyranoside | ^[5][a]^ |
|  |  | quercetin-3-O-α-L-rhamnopyranoside |  |
|  |  | quercetin-3-O-arabinoside-2’’-galloyl | ^[8]^ |
|  |  | quercetin-3-O-galactoside-2’’-galloyl |  |
|  |  | quercetin-hexoside (2) | ^[11][a]^ |
|  |  | quercetin 3-O-xyloside | ^[3]^ |
|  |  | quercetin hexose-galloyl (2) | ^[11][a]^ |
|  |  | quercetin-hexose-deoxyhexose-galloyl | ^[11][a]^ |
|  |  | quercetin-3-O-β-D-xylopyranoside (reynoutrin) | ^[5,9]^ |
|  |  | quercetin-3-rutinoside (rutin) | ^[1,3]^ |
|  |  | isorhamnetin 3-O-β-D-galactoside | ^[7]^ |
|  |  | isorhamnetin 3-O-β-D-rutinoside |  |
|  | Flavan-3-ols | catechin | ^[1,2,4,6,7,10]^ |
|  |  | epicatechin | ^[2,4,6,7,10]^ |
|  |  | epigallocatechin-3-gallate | ^[10]^ |
|  | Flavanones | hesperetin 7-O-rutinoside (hesperidin) | ^[3]^ |
| Non-flavonoid polyphenols | Gallotannins (hydrolysable tannins) | monogalloylquinic acid (4) | ^[8,11]^ |
|  |  | digalloylquinic acid (5) |  |
|  |  | trigalloylquinic acid (2) |  |
|  |  | tetragalloylquinic acid (5) |  |
|  |  | pentagalloylquinic acid (9) | ^[11][a]^ |
|  |  | 3,4-di-O-galloylquinic acid | ^[5][a]^ |
|  |  | 3,5-di-O-galloylquinic acid |  |
|  |  | 3,4,5-tri-O-galloylquinic acid |  |
|  |  | 1,3,4,5-tetra-O-galloylquinic acid |  |
|  | Phenolic acids | ferulic acid | ^[4,10]^ |
|  |  | gallic acid | ^[1,4,5,8–10]^ |
|  | Simple phenols | pyrogallol | ^[5][a]^ |
|  | Phenolic esters | methyl gallate | ^[5,8,9]^ |
|  |  | n-butil gallate | ^[5][a]^ |
| Triterpenoids | Lupanes | 2-β-hydroxy-lupeol | ^[6]^ |
|  |  | 2-α-hydroxy-lupeanolic acid | ^[7]^ |
|  |  | lupeanolic acid |  |
|  |  | betulin | ^[2]^ |
|  |  | betulinaldehyde |  |
|  |  | betulinic acid |  |
|  |  | lupeol | ^[2,6,12]^ |
|  | Oleananes | 2-α-hydroxy-oleanolic acid | ^[6,7]^ |
|  |  | 2-β-hydroxy-oleanolic acid |  |
|  |  | oleanolic acid | ^[2,6,7]^ |
|  |  | β-amyrin | ^[12][a]^ |
|  |  | β-amyrin acetate |  |
|  | Ursanes | bassic acid |  |
|  |  | α-amyrin |  |
|  |  | α-amyrin acetate |  |
|  |  | ursenaldehyde | ^[2]^ |
|  | Fitosterols | β-sitosterol |  |
|  |  | β-sitosterol glicoside (daucosterol) |  |
| Aminoacids | Common | aspartic acid |  |
|  |  | alanine |  |
|  |  | proline |  |
|  |  | valine |  |
|  | Non protein | 5-hydroxypipecolic acid |  |
|  |  | pipecolic acid |  |
| Glycolipids | Sulphoquinovosyl diacylglycerols | 1,2-di-O-palmitoyl-3-O-(6-sulpho-α-D-quinovopyranosyl)-glycerol | ^[2,6]^ |
|  |  | 1,2-di-O-(8-hexadecenoyl)-3-O-(6-sulpho-α-D-quinovopyranosyl)-glycerol | ^[6]^ |
|  |  | 1,2-di-O-miristoyl-3-O-(6-sulpho-α-D-quinovopyranosyl)-glycerol |  |
|  | Glucosyl diacylglycerols | 1,2-di-O-(8-hexadecenoyl)-3-O-(β-D-glucopyranosyl)-glycerol |  |
|  |  | 1,2-di-O-palmitoyl-3-O-(β-D-glucopyranosyl)-glycerol |  |
| Organic acids | Cyclohexanecarboxylic acids | quinic acid | ^[8]^ |
| Hydrocarbons | Straight-chain alkanes | dotriacontane | ^[12][a]^ |
| Fruits | | | |
| Flavonoids | Flavonols | galloylquercetin pentoside | ^[13]^ |
|  |  | quercetin deoxyhexosylhexoside |  |
|  |  | kaempferol | ^[14]^ |
|  |  | quercetin derivative |  |
|  |  | galloylquercetin hexoside | ^[13,14]^ |
|  |  | quercetin hexoside (3) |  |
|  |  | quercetin pentoside (3) |  |
|  |  | quercetin | ^[13–18]^ |
|  |  | quercetin-3-O-rutinoside (rutin) | ^[14,18]^ |
|  |  | quercetin-3-O-glucoside (isoquercitrin) | ^[18]^ |
|  |  | quercetin-O-hexoside |  |
|  |  | quercetin O-hexoside-deoxyhexoside |  |
|  |  | quercetin-O-pentoside |  |
|  |  | quercetin 3-O-pentoside |  |
|  | Flavones | apigenin | ^[16,18]^ |
|  |  | luteolin |  |
|  |  | luteolin 7-O-glucoside | ^[18]^ |
|  | Flavanonols | taxifolin | ^[14]^ |
|  | Flavan-3-ols | catechin | ^[14,18]^ |
|  |  | epicatechin | ^[14]^ |
|  | Proanthocyanidins (condensed tannins) | proanthocyanidin dimer (4) | ^[13,14,18]^ |
|  |  | galloylproanthocyanidin dimer | ^[13]^ |
|  |  | digalloylproanthocyanidin dimer |  |
| Non-flavonoid polyphenols | Gallotannins (hydrolysable tannins) | digalloylquinic acid (3) | ^[13,14]^ |
|  |  | tetragalloylquinic acid (2) |  |
|  |  | trigalloylquinic acid (3) |  |
|  |  | gallic acid derivative (4) |  |
|  |  | galloylquinic acid | ^[13]^ |
|  |  | digalloylglucose |  |
|  |  | digalloylpentose |  |
|  |  | digalloyl hexoside | ^[14]^ |
|  |  | digalloyl pentoside |  |
|  |  | galloylglucose isomer | ^[18]^ |
|  | Ellagitannins (hydrolyzable tannins) | ellagic acid galloyl hexoside | ^[14]^ |
|  | Phenolic acids | gallic | ^[13–18]^ |
|  |  | caffeic | ^[16,18]^ |
|  |  | syringic | ^[16,17]^ |
|  |  | p-coumaric |  |
|  |  | ferulic | ^[16]^ |
|  |  | ferulic-O-hexoside | ^[18]^ |
|  |  | protocatechuic acid |  |
|  |  | vanillic |  |
|  | Phenolic esters | ethyl gallate | ^[16,18]^ |
|  | Simple phenols | 3-methoxy-4-hydroxyphenol-1-O-β-D-(6'-O-galloyl)-glucoside | ^[18]^ |
| Tetraterpenes | Xanthophylls and esters | lutein (5) | ^[19–22]^ |
|  |  | lutein dilaurate | ^[20]^ |
|  |  | lutein 3'-O-laurate-3-O-myristate |  |
|  |  | lutein dimyristate |  |
|  |  | lutein 3'-O-palmitate-3-O-myristate |  |
|  |  | zeinoxanthin | ^[21,22]^ |
|  |  | zeaxanthin | ^[19,21,22]^ |
|  |  | β-cryptoxanthin |  |
|  | Carotenes | phytoene | ^[19]^ |
|  |  | phytofluene |  |
|  |  | α-carotene | ^[19,21,22]^ |
|  |  | β-carotene (3) | ^[19–22]^ |
| Carboxylic acids | Esters | methyl hexanoate | ^[16,23]^ |
|  |  | ethyl acetate | ^[24]^ |
|  |  | methyl octanoate |  |
|  |  | ethyl hexanoate | ^[23,24]^ |
|  |  | ethyl octanoate |  |
|  |  | ethyl 2-hydroxypropanoate | ^[23]^ |
|  |  | ethyl 3-methylthiopropanoate |  |
|  |  | ethyl butanoate |  |
|  |  | ethyl decanoate |  |
|  |  | ethyl hexadecanoate |  |
|  |  | methyl methylthiopropanoate |  |
|  |  | ethyl octadecenoate |  |
|  |  | methyl octadienoate |  |
|  | Free | hexanoic | ^[16,23,24]^ |
|  |  | hexadecanoic | ^[23]^ |
|  |  | butanoic |  |
|  |  | decanoic |  |
|  |  | octanoic |  |
| Alcohols | Aliphatics | 1-octen-3-ol | ^[23]^ |
|  |  | 2-methyl-1-propanol |  |
|  |  | 3-methyl-1-butanol |  |
|  |  | phenethyl alcohol |  |
|  |  | methionol |  |
| Lactones | δ-decalactone | δ-decalactone | ^[23]^ |
| Carboxylic acids | Tricarboxylic acids | citric acid | ^[16,25,26]^ |
|  | Ketoaldonic acids | ascorbic acid | ^[13,15,25–32]^ |
| Bark | | | |
| Flavonoids | Flavan-3-ols | epicatechin | ^[33–35]^ |
|  |  | catechin |  |
|  |  | epicatechin-3 gallate | ^[33]^ |
|  | Proanthocyanidins (condensed tannins) | (-)-epicatechin-[4α→8]-(-)-epicatechin | ^[34]^ |
|  |  | (+)-epicatechin-[4α→6]-(+)-epicatechin | ^[35]^ |
|  |  | (+)-epicatechin-[4α→8]-(+)-epicatechin |  |
|  |  | (+)-epicatechin-[4α→8]-3-O-galloyl-(+)-epicatechin |  |
|  |  | 3-O-galloyl-(-)-epicatechin-[4β→8]-3-O-galloyl-(+)-epicatechin-[4α→8]-(+)-epicatechin |  |
|  |  | 3-O-galloyl-(+)-epicatechin-[4α→8]-(+)-epicatechin |  |
|  |  | 3-O-galloyl-(+)-epicatechin-[4α→8]-3-O-galloyl-(+)-epicatechin |  |
|  |  | 3-O-galloyl-(+)-epicatechin-[4α→8]-3-O-galloyl-(+)-epicatechin-[4α→8]-(+)-epicatechin |  |
| Non-flavonoid polyphenols | Gallotannins (hydrolysable tannins) | 3-O-galloylquinic acid | ^[33]^ |
|  |  | 5-O-galloylquinic acid |  |
|  |  | 3,4-di-O-galloylquinic acid |  |
|  |  | 3,5-di-O-galloylquinic acid |  |
|  |  | 3,4,5-tri-O-galloylquinic acid |  |
|  | Phenolic acids | 3-O-caffeoylquinic acid | ^[33]^ |
|  |  | gallic acid | ^[34,35]^ |
|  |  | protocatechuic acid | ^[34]^ |
| Triterpenoids | Oleananes | β-amyrine | ^[36]^ |
| Seeds | | | |
| Fatty acids | Unsaturated | oleic | ^[23]^ |
|  |  | linoleic |  |
|  |  | palmitic |  |
|  | Saturated | butyric |  |
|  |  | hexanoic |  |
|  |  | stearic |  |
|  |  | lauric |  |
|  |  | myristic |  |
|  |  | palmitoleic |  |
| Diterpenoids | Labdanes | labda-17-acetoxi-13E-en-15-palmitate | ^[37]^ |
|  |  | labda-8(17),13E-dien-19-carboxy-15-il palmitate |  |
| Sesquiterpenoids | Lactones | byrsonina A | ^[38]^ |
|  |  | byrsonina B |  |
| Triterpenoids | Oleananes | birsonimadiol | ^[39]^ |
| Flowers | | | |
| Fatty acids | Saturated | stearic | ^[40]^ |
|  |  | myristic |  |
|  |  | palmitic |  |
|  | Unsaturated | C22 unknow |  |
|  |  | eicosadienoic |  |
|  |  | linoleic |  |
|  |  | oleic |  |
| Numbers in parentheses indicate the number of putatively identified isomers for a given compound.  [a] Plant material reported as *Byrsonima fagifolia* Nied. in the original study, a taxonomic synonym of *Byrsonima crassifolia* (L.) Kunth. | | | |

**References**

[1]	M. A. Cavalcante, J. S. Oliveira, M. S. S. Barreto, L. P. Pinheiro, P. C. Cantuária, W. L. Borges, G. A. Silva, T. M. Souza, “An HPLC Method to Determine Phenolic Compounds of Plant Extracts: Application to Byrsonima crassifolia and Senna alata Leaves” *Pharmacognosy Res.* **2022**, *14*, 395–404.

[2]	E. Béjar, A. Amarquaye, C. T. Che, M. H. Malone, H. H. S. Fong, “Constituents of Byrsonima crassifolia and their Spasmogenic Activity” *International Journal of Pharmacognosy* **1995**, *33*, 25–32.

[3]	M. Herrera-Ruiz, A. Zamilpa, M. González-Cortazar, R. Reyes-Chilpa, E. León, M. P. García, J. Tortoriello, M. Huerta-Reyes, “Antidepressant effect and pharmacological evaluation of standardized extract of flavonoids from Byrsonima crassifolia” *Phytomedicine* **2011**, *18*, 1255–1261.

[4]	V. H. A. Do Nascimento, A. Carla, G. Sobrinho, ; Cintya, O. Souza, J. Nazareno, S. De Souza, ; Consuelo, L. Sousa, “Determination of Phenolic Compounds with Antimicrobial Activity of Byrsonima Crassifolia and Inga Edulis Leaves Extracts” *Ensaios e Ciência: Ciências Biológicas, Agrárias e da Saúde* **2021**, *25*, 21–28.

[5]	M. Sannomiya, C. M. Rodrigues, G. C. A. Oliveira, J. C. S. Carvalho, L. S. da Costa, C. de C. Spadari, M. J. P. Ferreira, W. Vilegas, K. Ishida, “Galloylquinic acid derivatives from Byrsonima fagifolia leaf extract and potential antifungal activity” *J. Ethnopharmacol.* **2022**, *297*, 115534.

[6]	L. Rastrelli, N. De Tommasi, I. Berger, A. Caceres, A. Saravia, F. De Simone, “Glycolipids from Byrsonima crassifolia” *Phytochemistry* **1997**, *45*, 647–650.

[7]	I. Berger, A. C. Barrientos, A. Cáceres, M. Hernández, L. Rastrelli, C. M. Passreiter, W. Kubelka, “Plants used in Guatemala for the treatment of protozoal infections” *J. Ethnopharmacol.* **1998**, *62*, 107–115.

[8]	A. F. L. Specian, K. Tuttis, J. M. Serpeloni, D. L. Ribeiro, H. L. Nunes, M. M. P. Tangerina, M. Sannomiya, E. A. Varanda, W. Vilegas, I. M. S. Cólus, “Chemical characterization of Brazilian savannah Byrsonima species (muricis) and their impact on genomic instability and chemopreventive effects” *Mutation Research/Genetic Toxicology and Environmental Mutagenesis* **2023**, *887*, 503586.

[9]	Z. P. Lima, R. de C. dos Santos, T. U. Torres, M. Sannomiya, C. M. Rodrigues, L. C. dos Santos, C. H. Pellizzon, L. R. M. Rocha, W. Vilegas, A. R. M. Souza Brito, C. R. P. Cardoso, E. A. Varanda, H. P. de Moraes, T. M. Bauab, C. Carli, I. Z. Carlos, C. A. Hiruma-Lima, “Byrsonima fagifolia: An integrative study to validate the gastroprotective, healing, antidiarrheal, antimicrobial and mutagenic action” *J. Ethnopharmacol.* **2008**, *120*, 149–160.

[10]	R. O. de Souza, G. de A. D. Alves, A. L. S. A. Forte, F. Marquele-Oliveira, D. F. da Silva, H. Rogez, M. J. V. Fonseca, “Byrsonima crassifolia extract and fraction prevent UVB-induced oxidative stress in keratinocytes culture and increase antioxidant activity on skin” *Ind. Crops Prod.* **2017**, *108*, 485–494.

[11]	M. Sannomiya, L. C. Dos Santos, V. Carbone, A. Napolitano, S. Piacente, C. Pizza, A. R. M. Souza-Brito, W. Vilegas, “Liquid chromatography/electrospray ionization tandem mass spectrometry profiling of compounds from the infusion of Byrsonima fagifolia Niedenzu” *Rapid Communications in Mass Spectrometry* **2007**, *21*, 1393–1400.

[12]	C. T. Higuchi, M. Sannomiya, F. R. Pavan, S. R. A. Leite, DN. Sato, S. G. Franzblau, L. V. S. Sacramento, W. Vilegas, C. Q. F. Leite, “Byrsonima fagifolia niedenzu apolar compounds with antitubercular activity” *Evidence-based Complementary and Alternative Medicine* **2011**, *2011*, DOI 10.1093/ECAM/NEN077.

[13]	A. Gordon, E. Jungfer, B. A. Da Silva, J. G. S. Maia, F. Marx, “Phenolic Constituents and Antioxidant Capacity of Four Underutilized Fruits from the Amazon Region” *J. Agric. Food Chem.* **2011**, *59*, 7688–7699.

[14]	L. R. B. Mariutti, E. Rodrigues, R. C. Chisté, E. Fernandes, A. Z. Mercadante, “The Amazonian fruit Byrsonima crassifolia effectively scavenges reactive oxygen and nitrogen species and protects human erythrocytes against oxidative damage” *Food Research International* **2014**, *64*, 618–625.

[15]	M. S. B. Sousa, I. M. S. de Holanda, H. M. C. Monteiro, Â. Amâncio-Dos-Santos, “Antioxidant extract counteracts the effects of aging on cortical spreading depression and oxidative stress in the brain cortex” *Acta Cir. Bras.* **2018**, *33*, 472–482.

[16]	J. V. Faria, I. H. Valido, W. H. P. Paz, F. M. A. da Silva, A. D. L. de Souza, L. R. D. Acho, E. S. Lima, A. P. A. Boleti, J. V. N. Marinho, M. J. Salvador, E. L. dos Santos, P. K. Soares, M. López-Mesas, J. M. F. Maia, H. H. F. Koolen, G. A. Bataglion, “Comparative evaluation of chemical composition and biological activities of tropical fruits consumed in Manaus, central Amazonia, Brazil” *Food Research International* **2021**, *139*, 109836.

[17]	K. S. L. Miki, A. P. Dresch, M. Cavali, A. P. da Silva, F. Marafon, O. Fogolari, G. M. Mibielli, M. D. Bagatini, J. P. Bender, “Influence of drying methods in the ultrasound-assisted extraction of bioactive compounds from Byrsonima crassifolia to evaluate their potential antitumor activity” *Food and Humanity* **2024**, *2*, 100242.

[18]	T. P. B. Lima, P. P. S. Coimbra, A. da Silva Antonio, H. M. G. Pereira, G. R. P. da Silva, V. F. da Veiga-Junior, O. F. Silva, I. Felzenszwalb, C. F. Araujo-Lima, A. J. Teodoro, “Antioxidant activity, phytochemical composition, and antitumor capacity of Amazonian fruits taperebá (Spondias mombin) and murici (Byrsonima crassifolia)” *Arch. Pharm. (Weinheim).* **2025**, *358*, e2400758.

[19]	L. R. B. Mariutti, E. Rodrigues, A. Z. Mercadante, “Carotenoids from Byrsonima crassifolia: Identification, quantification and in vitro scavenging capacity against peroxyl radicals” *Journal of Food Composition and Analysis* **2013**, *31*, 155–160.

[20]	A. Irías-Mata, V. M. Jiménez, C. B. Steingass, R. M. Schweiggert, R. Carle, P. Esquivel, “Carotenoids and xanthophyll esters of yellow and red nance fruits (Byrsonima crassifolia (L.) Kunth) from Costa Rica” *Food Research International* **2018**, *111*, 708–714.

[21]	V. R. De Souza, M. C. M. Brum, I. D. S. Guimarães, P. De Freitas Dos Santos, T. O. Do Amaral, J. P. Abreu, T. Passos, O. Freitas-Silva, E. R. P. Gimba, A. J. Teodoro, “Amazon Fruits Inhibit Growth and Promote Pro-apoptotic Effects on Human Ovarian Carcinoma Cell Lines” *Biomolecules 2019, Vol. 9, Page 707* **2019**, *9*, 707.

[22]	R. M. Martinez, A. C. Cintra, C. de O. R. P. de Almeida, T. P. B. Lima, I. Moraes de Souza, M. A. P. de Almeida, S. P. Frankenfeld, F. L. C. Cahuê, G. de Queiroz Pereira Lopes, V. S. Pinto, A. R. Silva, C. F. Gonçalves-De-Albuquerque, M. Dolisnky, A. J. Teodoro, “Bioactive Properties of Murici (Byrsonima crassifolia) and Bacuri (Platonia insignis) and Their Antioxidant and Anti-Inflammatory Effects in Rats” *https://home.liebertpub.com/jmf* **2025**, *28*, 478–489.

[23]	C. M. Rezende, S. R. G. Fraga, “Chemical and aroma determination of the pulp and seeds of murici (Byrsonima crassifolia L.)” *J. Braz. Chem. Soc.* **2003**, *14*, 425–428.

[24]	A. P. G. da Silva, P. C. Spricigo, E. Purgatto, S. M. de Alencar, A. P. Jacomino, “Volatile Compounds Determined by SPME-GC, Bioactive Compounds, In Vitro Antioxidant Capacity and Physicochemical Characteristics of Four Native Fruits from South America” *Plant Foods Hum. Nutr.* **2019**, *74*, 358–363.

[25]	V. R. De Souza, P. A. P. Pereira, F. Queiroz, S. V. Borges, J. De Deus Souza Carneiro, “Determination of bioactive compounds, antioxidant activity and chemical composition of Cerrado Brazilian fruits” *Food Chem.* **2012**, *134*, 381–386.

[26]	A. Aniceto, J. Montenegro, R. da S. Cadena, A. J. Teodoro, “Physicochemical Characterization, Antioxidant Capacity, and Sensory Properties of Murici (Byrsonima crassifolia (L.) Kunth) and Taperebá (Spondias mombin L.) Beverages” *Molecules 2021, Vol. 26, Page 332* **2021**, *26*, 332.

[27]	M. S. B. Sousa, D. V. de S. Alves, H. M. C. Monteiro, D. A. Gomes, E. C. Lira, A. Amancio-dos-Santos, “Sepsis impairs the propagation of cortical spreading depression in rats and this effect is prevented by antioxidant extract” *Nutr. Neurosci.* **2019**, *24*, 130–139.

[28]	M. S. M. Rufino, F. A. N. Fernandes, R. E. Alves, E. S. de Brito, “Free radical-scavenging behaviour of some north-east Brazilian fruits in a DPPH system” *Food Chem.* **2009**, *114*, 693–695.

[29]	G. A. B. Canuto, A. A. O. Xavier, C. N. Leandro, M. T. de Benassi, “Caracterização físico-química de polpas de frutos da Amazônia e sua correlação com a atividade anti-radical livre” *Rev. Bras. Frutic.* **2010**, *32*, 1196–1205.

[30]	M. M. B. Almeida, P. H. M. de Sousa, Â. M. C. Arriaga, G. M. do Prado, C. E. de C. Magalhães, G. A. Maia, T. L. G. de Lemos, “Bioactive compounds and antioxidant activity of fresh exotic fruits from northeastern Brazil” *Food Research International* **2011**, *44*, 2155–2159.

[31]	R. S. D. R. Moreira-Araújo, N. V. D. A. Barros, R. G. C. L. Porto, A. de C. A. S. Brandão, A. de Lima, R. Fett, “Bioactive compounds and antioxidant activity three fruit species from the Brazilian Cerrado” *Rev. Bras. Frutic.* **2019**, *41*, e-011.

[32]	P. R. S. Borges, M. Edelenbos, E. Larsen, T. Hernandes, E. E. Nunes, E. V. de Barros Vilas Boas, C. R. F. Pires, “The bioactive constituents and antioxidant activities of ten selected Brazilian Cerrado fruits” *Food Chem. X* **2022**, *14*, 100268.

[33]	M. Maldini, P. Montoro, C. Pizza, “Phenolic compounds from Byrsonima crassifolia L. bark: Phytochemical investigation and quantitative analysis by LC-ESI MS/MS” *J. Pharm. Biomed. Anal.* **2011**, *56*, 1–6.

[34]	M. de la Cabeza Fernández, M. Sánchez, A. Caceres, I. Iglesias, M. P. Gómez-Serranillos, “Neuropharmacological Effects in Animal Models and HPLC-Phytochemical Profiling of Byrsonima crassifolia (L.) Kunth Bark Extracts” *Molecules 2023, Vol. 28, Page 764* **2023**, *28*, 764.

[35]	F. Geiss, M. Heinrich, D. Hunkler, H. Rimpler, “Proanthocyanidins with ( + )-epicatechin units from Byrsonima crassifolia bark” *Phytochemistry* **1995**, *39*, 635–643.

[36]	C. Djerassi, A. Bowers, S. Burstein, B. Estrada, J. Grossman, J. Herrán, A. J. Lemin, A. Manjarrez, S. C. Pakrashi, “Terpenoids. XXII. Triterpenes from Some Mexican and South American Plants” *J. Am. Chem. Soc.* **1956**, *78*, 2312–2315.

[37]	A. Muñiz-Ramirez, R. M. Perez-Gutiérrez, E. Garcia-Baez, J. M. Mota-Flores, “Antimicrobial activities of diterpene labdane from seeds of Byrsonima crassifolia” *Bol. Latinoam. Caribe Plantas Med. Aromat.* **2014**, *13*, 31–37.

[38]	R. M. P. Gutiérrez, A. M. Ramirez, “Hypoglycemic Effects of sesquiterpene lactones from Byrsonima crassifolia.” *Food Sci. Biotechnol.* **2016**, *25*, 1135–1145.

[39]	R. M. P. Gutiérrez, “Anti-inflammatory Effect of Birsonimadiol from Seeds of Byrsonima crassifolia” *Food Sci. Biotechnol* **2016**, *25*, 561–566.

[40]	S. B. Vinson, H. J. Williams, G. W. Frankie, G. Shrum, “Floral Lipid Chemistry of Byrsonima crassifolia (Malpigheaceae) and a Use of Floral Lipids by Centris Bees (Hymenoptera: Apidae)” *Biotropica* **1997**, *29*, 76–83.
